# Reconstructing missing time-varying land subsidence data using back propagation neural network with principal component analysis

**DOI:** 10.1038/s41598-023-44642-1

**Published:** 2023-10-13

**Authors:** Chih-Yu Liu, Cheng-Yu Ku, Jia-Fu Hsu

**Affiliations:** 1 Department of Civil Engineering, National Central University, Taoyuan, 320317 Taiwan; 2 Department of Harbor and River Engineering, National Taiwan Ocean University, Keelung, 20224 Taiwan

**Keywords:** Environmental sciences, Hydrology, Natural hazards, Engineering

## Abstract

Land subsidence, a complex geophysical phenomenon, necessitates comprehensive time-varying data to understand regional subsidence patterns over time. This article focuses on the crucial task of reconstructing missing time-varying land subsidence data in the Choshui Delta, Taiwan. We propose a novel algorithm that leverages a multi-factorial perspective to accurately reconstruct the missing time-varying land subsidence data. By considering eight influential factors, our method seeks to capture the intricate interplay among these variables in the land subsidence process. Utilizing Principal Component Analysis (PCA), we ascertain the significance of these influencing factors and their principal components in relation to land subsidence. To reconstruct the absent time-dependent land subsidence data using PCA-derived principal components, we employ the backpropagation neural network. We illustrate the approach using data from three multi-layer compaction monitoring wells from 2008 to 2021 in a highly subsiding region within the study area. The proposed model is validated, and the resulting network is used to reconstruct the missing time-varying subsidence data. The accuracy of the reconstructed data is evaluated using metrics such as root mean square error and coefficient of determination. The results demonstrate the high accuracy of the proposed neural network model, which obviates the need for a sophisticated hydrogeological numerical model involving corresponding soil compaction parameters.

## Introduction

Land subsidence, a gradual settling of the ground surface over extended time periods, has been extensively studied^1–3^. Land subsidence is a geological phenomenon characterized by the downward movement of the ground. Natural causes of land subsidence include the compaction of sediment layers, which can be referred to Terzaghi consolidation theory. It is well known that the process of soil consolidation, which is the gradual settlement and compression of soils as water is expelled from their pores. The intrinsic factors such as the sediment type and drainage path length could potentially impact land subsidence in the Terzaghi consolidation theory. On the other hand, the extrinsic factors such as human activities induced groundwater level variation, pumping and intensified agricultural activity are often the primary contributors to accelerated subsidence. The precipitation may also be an important factor for recharging the groundwater affecting the subsidence.

The availability of complete time-varying land subsidence data is crucial for capturing the spatio-temporal characteristics of regional subsidence, especially in the context of global climate change^4,5^. Understanding the compression of soil strata resulting from groundwater level fluctuations is an essential aspect of subsidence analysis^6–9^. In the Choshui Delta, Taiwan, regional subsidence has been systematically monitored for approximately 15 years^10–14^. However, to investigate the impact of climate change on land subsidence, long-term decadal time series are required, prompting initiatives focused on reconstructing such data^15,16^.

Machine learning techniques, such as neural networks, have garnered significant attention in the geosciences, particularly for predicting groundwater fluctuations. Neural networks possess the capability to reconstruct missing data by utilizing interconnected matrices of bias and weight within the neurons of hidden layers^17–19^. During the training process, weights and biases are optimized to align the network's response with the training data output. Subsequently, validation is performed to assess the network's generalization, which relies on the quality and quantity of training data, as well as the network architecture^20^. Conventional numerical modeling approaches for land subsidence, which rely on the physical mechanisms, often necessitate sophisticated three-dimensional models^21–24^. Additionally, reliable hydrogeological parameters for the soil's physical properties are crucial^25,26^. However, acquiring these parameters is challenging due to spatial variations in soil strata across different regions. In light of these challenges, neural network methods offer promising alternatives, particularly when time-dependent observations and monitoring data are available^27–29^. These methods can overcome the limitations of conventional modeling approaches by leveraging the power of data-driven learning algorithms.

In this study, we propose a novel algorithm that leverages a multi-factorial perspective to accurately reconstruct the missing time-varying land subsidence data. By considering eight influential factors, our method seeks to capture the intricate interplay among these variables in the land subsidence process. Utilizing Principal Component Analysis (PCA), we ascertain the significance of these influencing factors and their principal components in relation to land subsidence. To reconstruct the absent time-dependent land subsidence data using PCA-derived principal components, we employ the backpropagation neural network. We illustrate the approach using data from three multi-layer compaction monitoring wells from 2008 to 2021 in a highly subsiding region within the study area. The proposed model is validated, and the resulting network is used to reconstruct the missing time-varying subsidence data.

## Study area and datasets

### Study area

**Figure 1:**
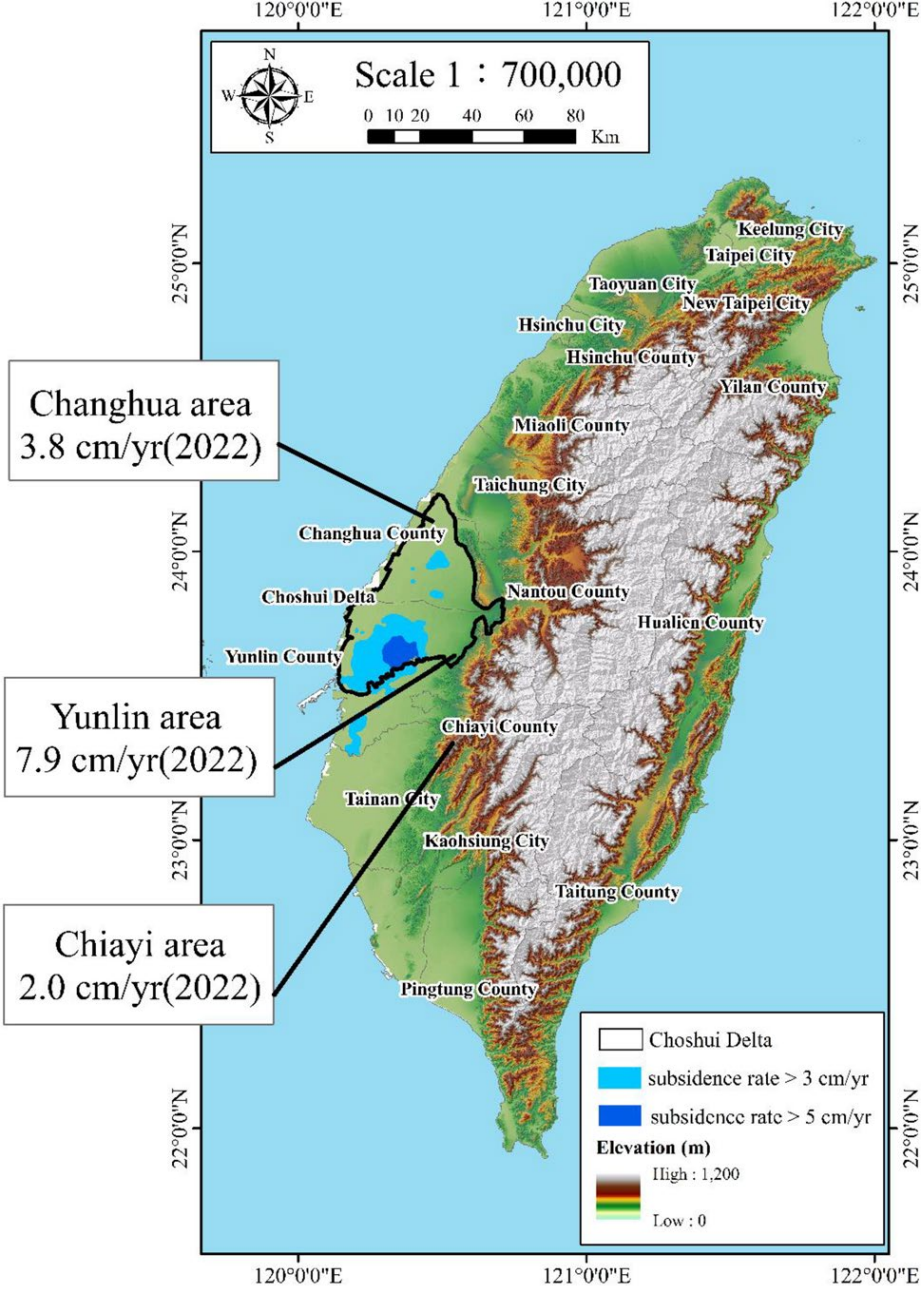
Significant subsidence areas in Taiwan in 2022. This figure was created using ArcGIS 10.6.1 software.

During the 1970s, researchers noted instances of subsidence along the southern coastal regions of the Choshui Delta located on the west coast of central Taiwan^8,12,13^. This phenomenon escalated in severity, resulting in detrimental effects on public infrastructure and various other issues. Although subsidence in coastal areas has witnessed a deceleration over the past decade, it persists in inland regions. Presently, within the entirety of the delta, the central zone is experiencing the most significant rate of subsidence. According to the Water Resources Agency (WRA) under the Ministry of Economic Affairs of Taiwan, Yunlin County in the Choshui Delta registered the highest annual subsidence rate of 7.9 cm in 2022—a peak across Taiwan, as illustrated in Fig. 1.

**Figure 2:**
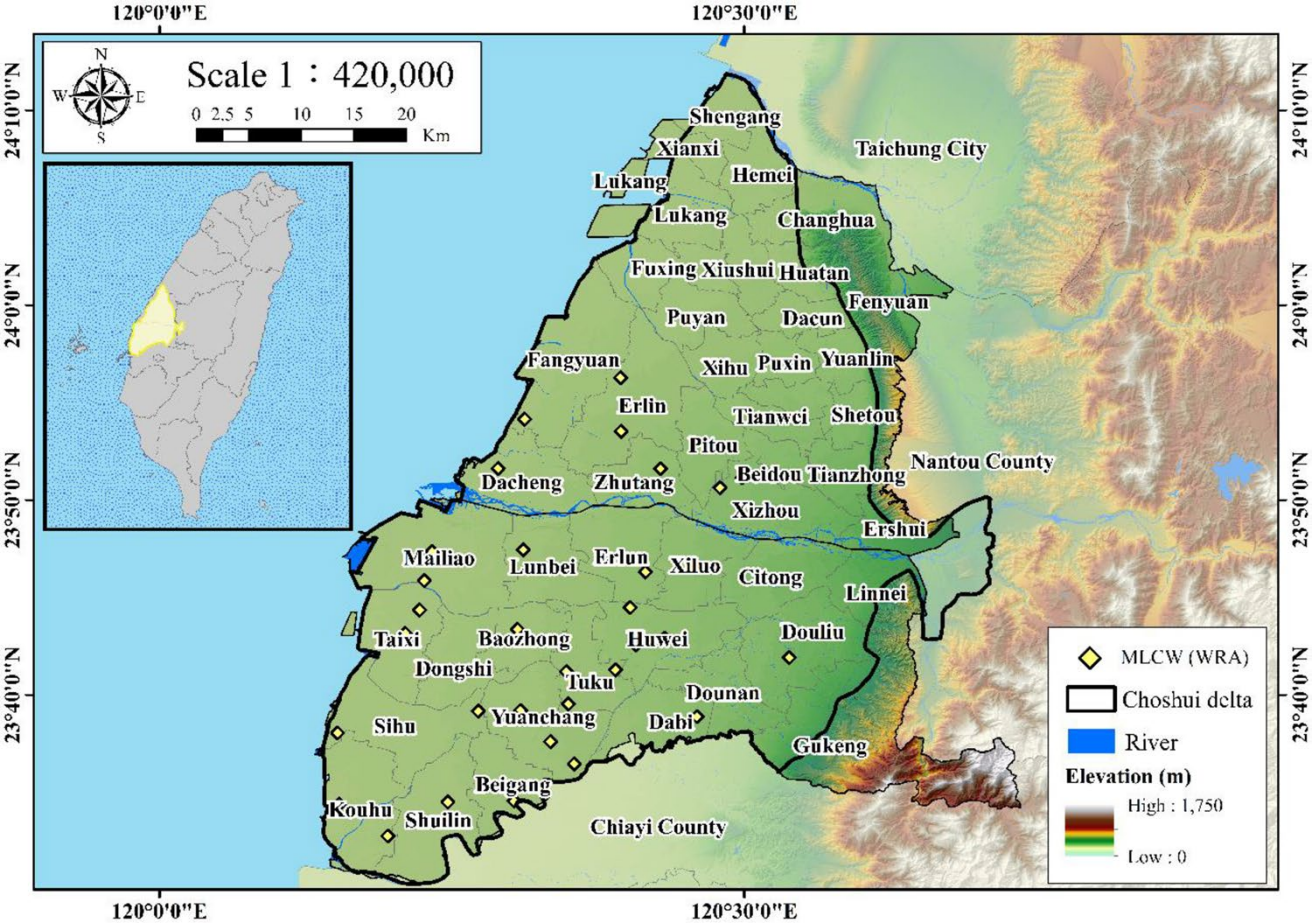
Location of the Choshui Delta. This figure was created using ArcGIS 10.6.1 software.

Notably, the most pronounced subsidence is prevalent in Tuku and Yuanchang Townships within the central Choshui Delta. Accordingly, the study area under investigation is the Choshui Delta, located in western Taiwan. The Choshui Delta encompasses an area of 2000 km^2^ with elevations ranging from 0 to 100 m (Fig. 2). The primary river, the Choshui River, originates from the western part of the central mountain range, flowing between the southern Hehuan Mountain and the northern side of Yushan Mountain. The Choshui Delta, known as an alluvial fan, is formed in the westward hilly region. The main river flows through the central part of the alluvial fan and eventually discharges into the Taiwan Strait.

**Figure 3:**
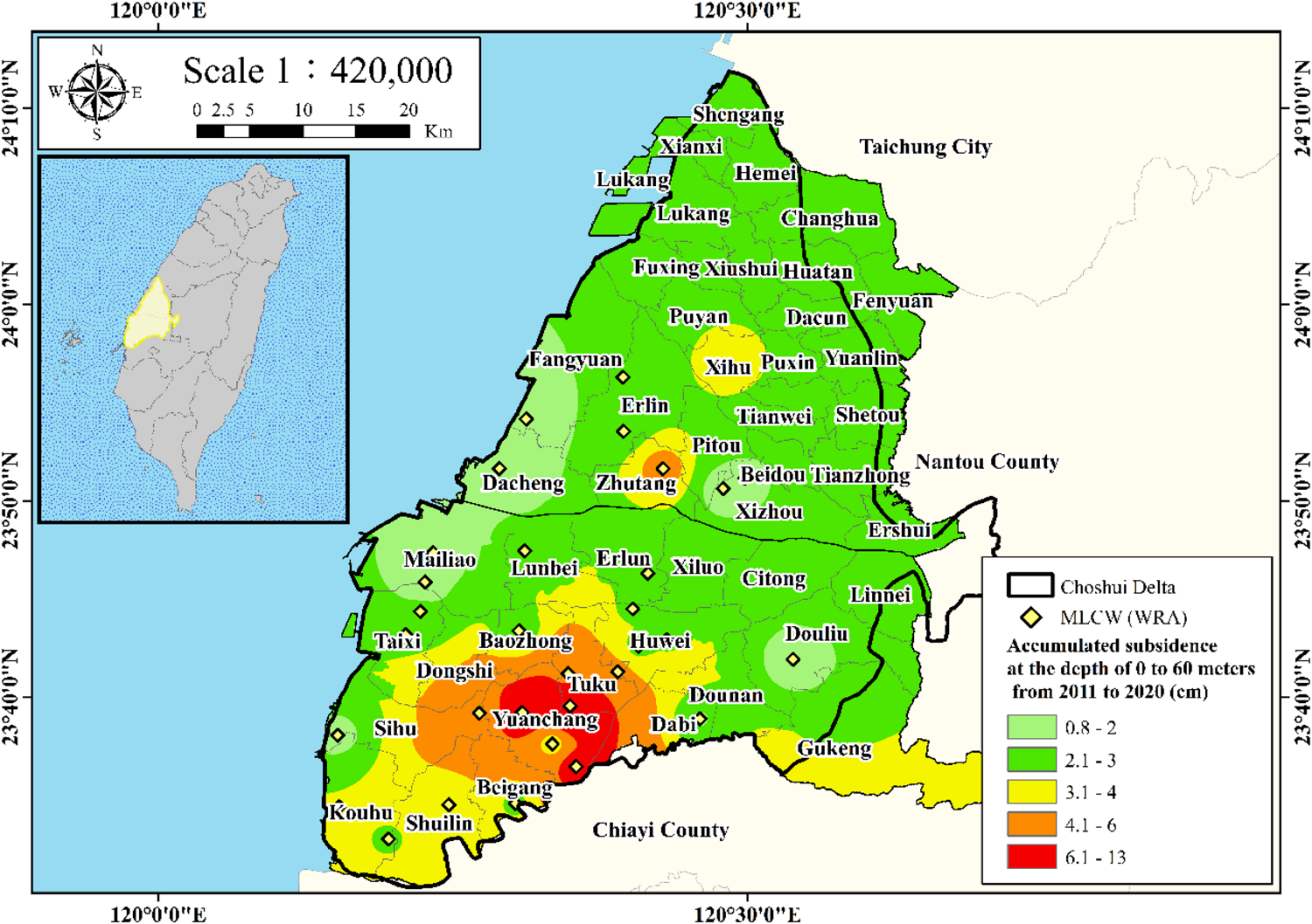
Accumulated subsidence from 2011 to 2020 at 0 to 60 m. This figure was created using ArcGIS 10.6.1 software.

Due to excessive groundwater extraction, the central area of the Choshui Delta faces significant land subsidence issues^8,12,13^. Figure 3 illustrates the accumulated subsidence from 2011 to 2020 in the depth range of 0 to 60 m. As shown in Fig. 3, the inland regions of Yunlin County contain the most severe subsidence areas, specifically in Huwei Township, Tuku Township, and Yuanchang Township. Consequently, Multi-Layer Compaction Wells (MLCWs) within these significant subsidence regions are selected for the application of the neural network to reconstruct the missing time-varying land subsidence data. The MLCW is a specialized monitoring instrument used to measure and assess land subsidence, particularly in areas where excessive groundwater extraction is a concern. MLCWs are designed with multiple sensors or observation points at different depths within the ground. These sensors record variations in the distance between them over time, allowing researchers to detect changes in the soil's compaction or compression at various depths. These MLCWs include Xiutan Elementary School (STES) in Tuku Township, Yuanchang Elementary School (YCES) in Yuanchang Township, and Neiliao Residency Station (NLPS) in Yuanchang Township.

### Datasets

**Figure 4:**
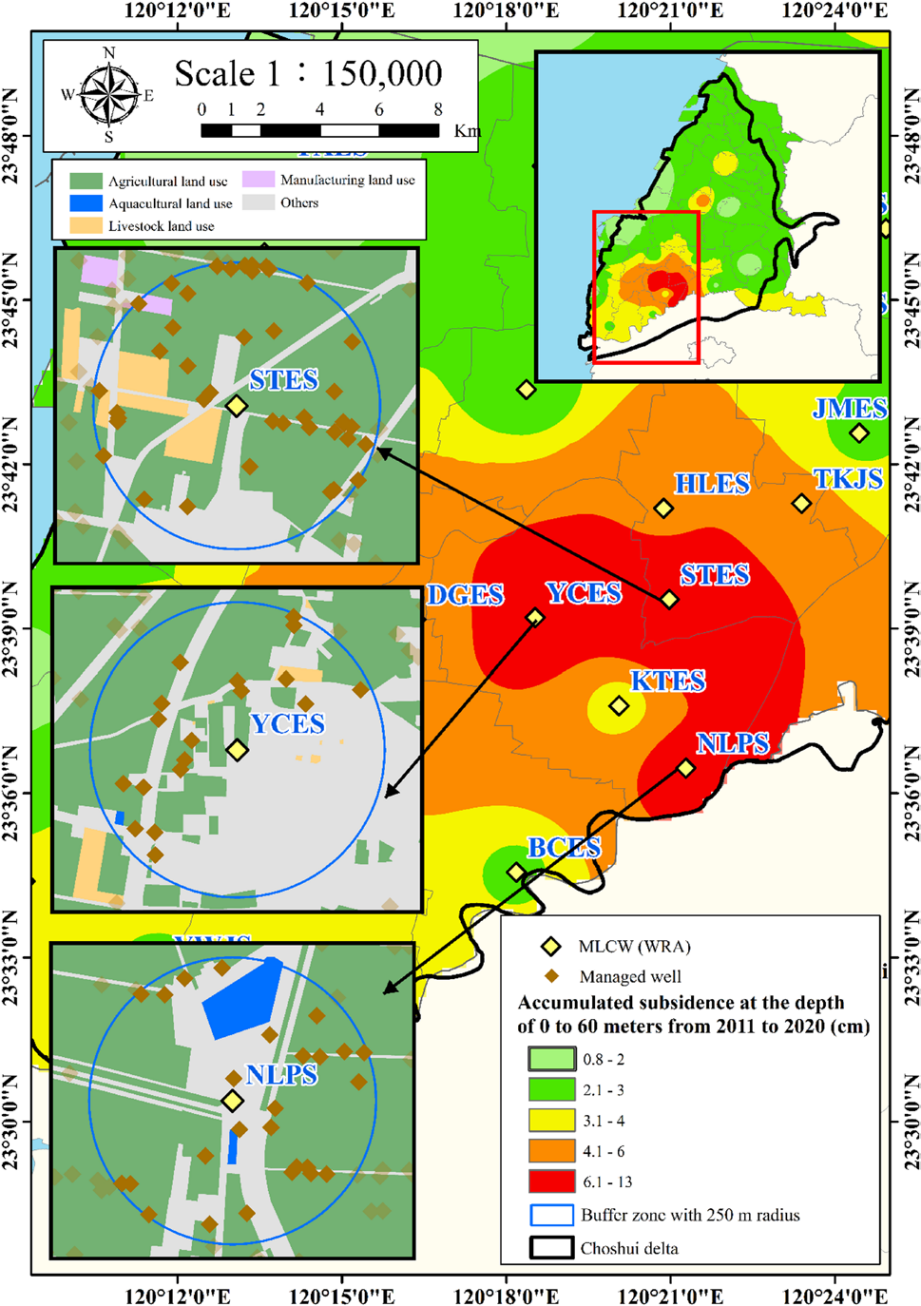
Location of multi-layer compaction monitoring wells. This figure was created using ArcGIS 10.6.1 software.

The geographical location of the study area, which includes STES, YCES, and NLPS, is depicted in Fig. 4. In this study, several time-dependent factors, including groundwater level data, electricity consumption data, and precipitation data are recognized as influential factors in land subsidence. These factors fall within the category of extrinsic factors associated with human activities. Monthly fluctuations in groundwater levels and electricity consumption (a proxy indicator for estimating groundwater usage) are typically the major contributors to accelerated subsidence. Furthermore, precipitation may also play a crucial role in recharging groundwater, which in turn impacts subsidence.

However, it is undeniable that, in addition to the factors mentioned above, other variables such as land use patterns, sediment type, and drainage path length could potentially impact land subsidence. For instance, intensified agricultural activity may result in land subsidence, particularly in regions with extensive irrigation practices. Fine-grained soils may be susceptible to land subsidence when subjected to excessive groundwater extraction. Thus, factors such as the percentage of fine-grained soil and the length of the average maximum drainage path may be considered relevant factors influencing land subsidence.

**Figure 5:**
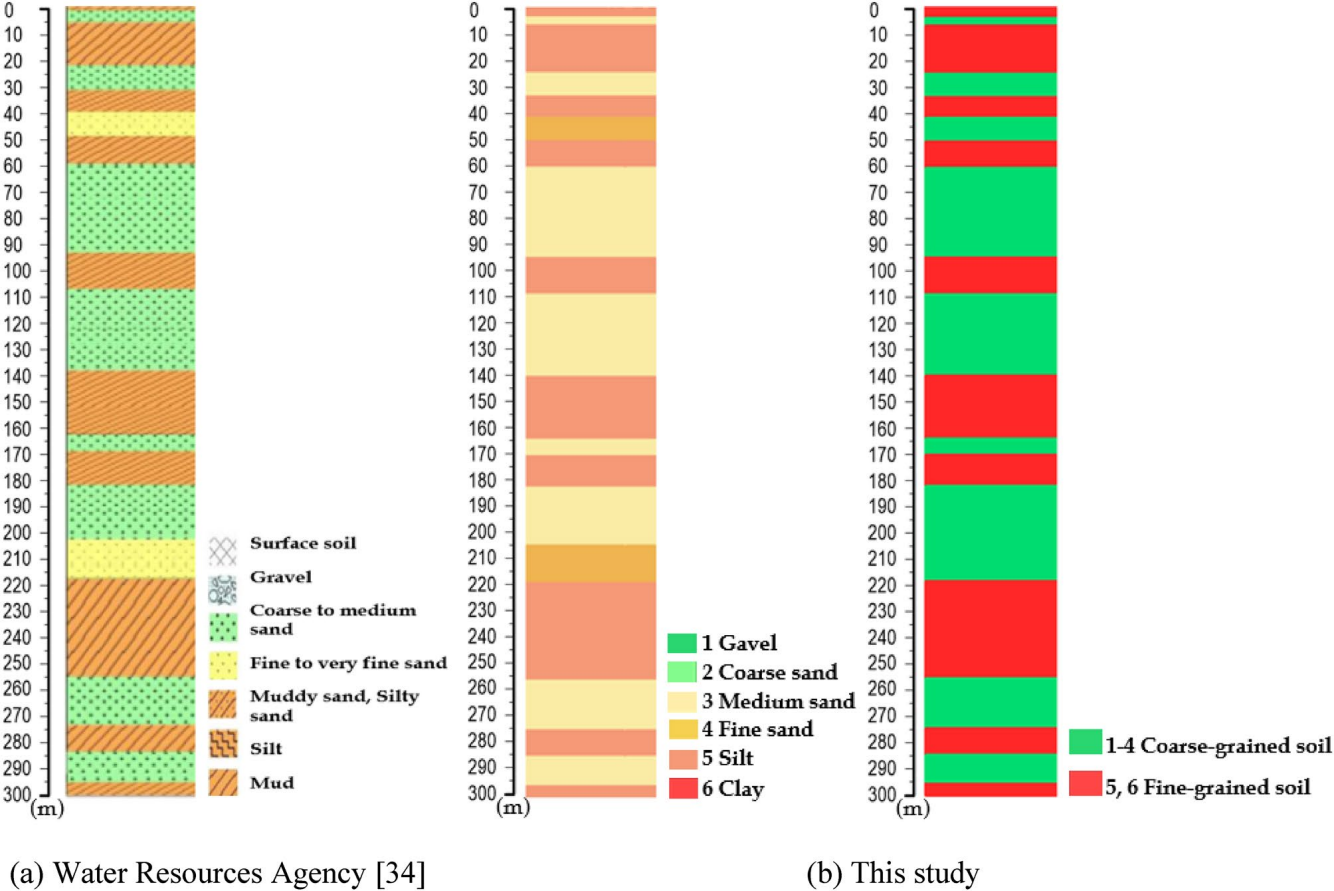
Examples of borehole logging data at STES.

Table 1 lists the source data utilized in this study. These datasets consist of the cumulative land subsidence data obtained from levelling surveys and MLCWs, groundwater level data, electricity consumption data, and precipitation data. The cumulative land subsidence data and groundwater level data are publicly accessible and sourced from the WRA, while electricity consumption data is also sourced from the WRA. Precipitation data is acquired from the Central Weather Bureau. The percentage of fine-grained soil and the length of the average maximum drainage path are derived from borehole logging data^30^, as shown in Fig. 5, provided by the Central Geological Survey (CGS) and WRA of Taiwan. The current state of land use, essential for calculating the percentage of agricultural land use, is obtained from the National Land Surveying and Mapping Center (NLSC), Ministry of the Interior. A detailed description of the datasets is provided below.

**Table 1:** Datasets in this study.

	Study area	Data availability of the source data			Data interval	Reference
		Xiutan elementary school (STES)	Yuanchang elementary school (YCES)	Neiliao residency station (NLPS)		
1	Cumulative land subsidence	January 2008 to November 2021 (Missing data: March 2012 to March 2014)			1 month	Multi-layer compaction monitoring wells (Water resources agency)
2	Groundwater level	January 2008 to December 2021			1 month	Multi-layer groundwater level monitoring wells (Water resources agency)
3	Electricity consumption of managed wells	January 2008 to December 2021			1 month	Managed wells (Water resources agency)
4	Average monthly precipitation	January 2008 to December 2021			1 day	Rainfall Gauges (Central Weather Bureau)
5	Percentage of agricultural land use	December 2021			NA	Land use data (National land surveying and mapping center)
6	Percentage of fine-grained soil	December 2021			NA	Borehole data (Central geological survey and water resources agency)
7	Length of average maximum drainage path	December 2021			NA	Borehole data (Central geological survey and water resources agency)

#### Monthly compaction change

Land subsidence can be primarily classified into three categories: subsidence resulting from groundwater extraction, subsidence triggered by the weight of structures, and subsidence caused by the natural consolidation of alluvial soil. Land subsidence datasets consist of the cumulative land subsidence data obtained from levelling surveys and MLCWs. Levelling surveys are a fundamental technique used in land surveying engineering to determine the relative elevations of different points on the Earth's surface. The MLCW technique is adopted to survey the compaction at different depth. The MLCW is a specialized monitoring instrument used to measure and assess land subsidence, particularly in areas where excessive groundwater extraction is a concern. The MLCW is designed with multiple sensors or observation points at different depths within the ground. These sensors record variations in the distance between them over time, allowing researchers to detect changes in the soil's compaction or compression at various depths. The primary purpose of MLCWs is to provide detailed and precise data on how land subsidence occurs at different layers beneath the surface. This information is crucial for understanding the subsidence process. MLCWs are valuable tools in regions prone to land subsidence, such as areas with excessive groundwater pumping or geological conditions that promote compaction of the subsurface materials.

The first MLCW of the subsidence network was carried out in 2008 and 31 MLCWs have been deployed in Choshui Delta since then^11,14^. The time varying subsidence data from the MLCW are crucial to precisely investigate the compression of the soil in spatial and temporal scale. The monitoring depth of the MLCW is ranging from 2.4 to 340 m. The variation observed between two neighboring rings depicts the deformation of the stratigraphic profile spanning between them. In the MLCW monitoring technique, rings refer to different sections or layers within the well that are instrumented to measure compaction at various depths. Each ring provides data on subsidence at a specific depth range. This information helps in understanding how subsidence varies with depth in the soil profile. The functioning of MLCWs involves measuring the compaction of these rings over time to monitor land subsidence. The compaction of each soil layer to the total subsidence is then measured. The MLCW has advantage of the monitoring subsidence with high accuracy of 1 mm^11^. The monthly compaction change is calculated as follows.

1 $$ \Delta C = C_{i} - C_{i - 1} , $$

where $\Delta C$ denotes the monthly compaction change, $C_{i}$ denotes the accumulated subsidence at the *i*-th month, and $C_{i - 1}$ denotes the accumulated subsidence at the (*i*–1)-th month.

**Figure 6:**
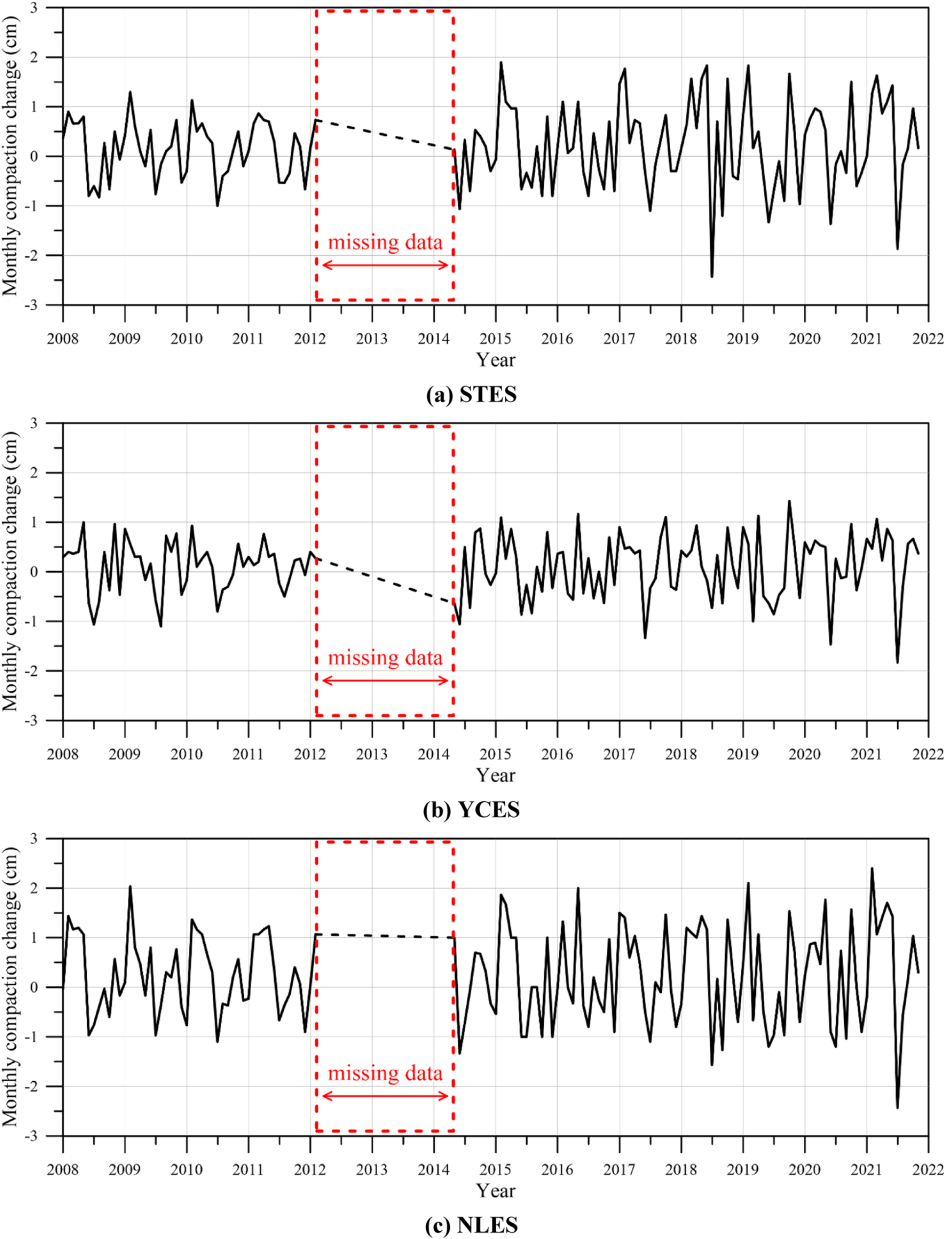
Plot of monthly compaction change versus year.

In this study, the MLCWs installed at STES, YCES, and NLPS are adopted because these areas are situated at the highest subsidence area, as shown in Fig. 4. The plot of monthly compaction change versus year at STES, YCES, and NLPS is demonstrated in Fig. 6a,b,c, respectively. It is found that the subsidence data from the MLCWs installed at STES, YCES, and NLPS are not available from 2012 to 2014. The missing time varying subsidence data will be reconstructed using the neural network in this study.

#### Monthly groundwater level variation

**Figure 7:**
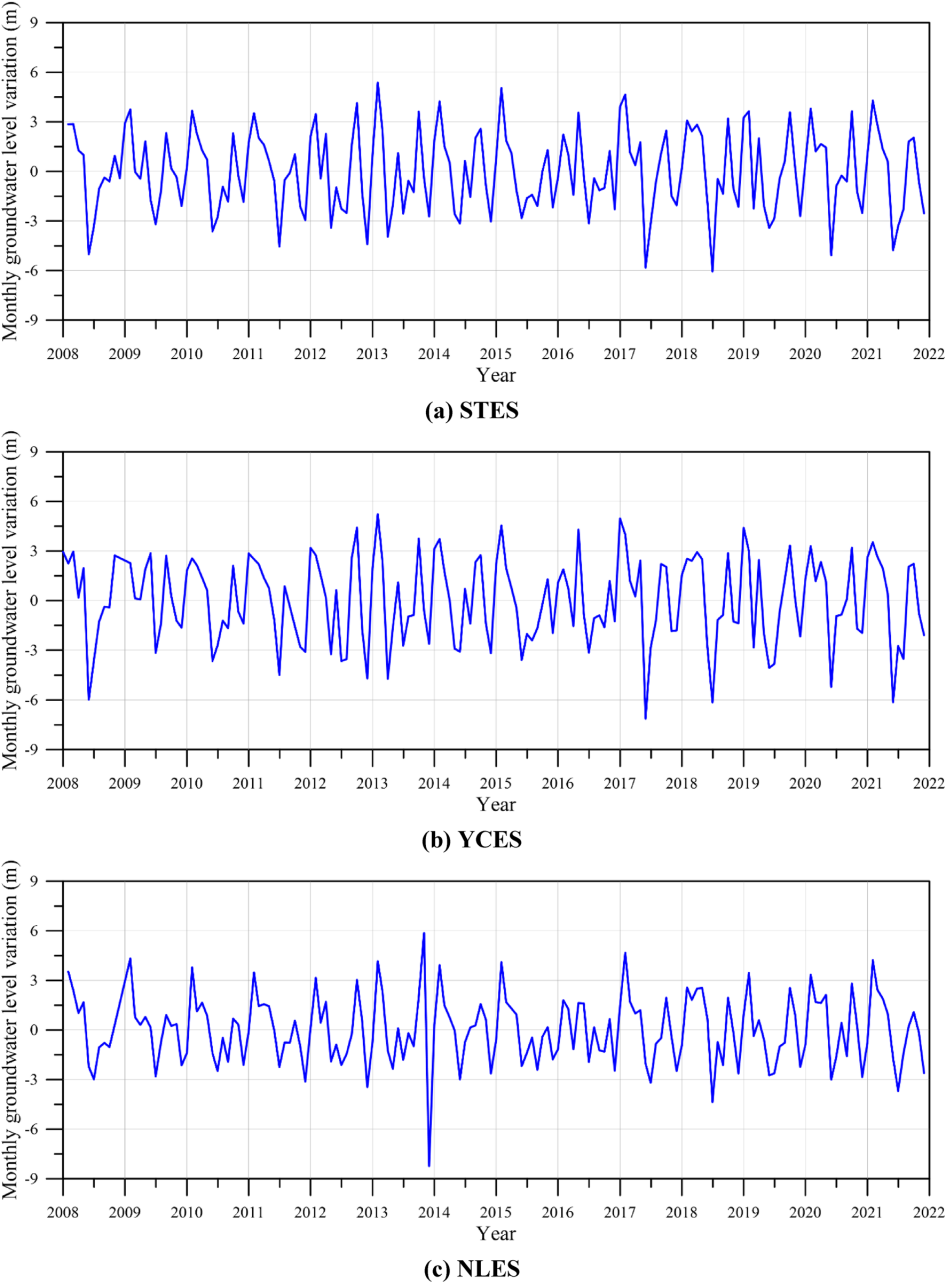
Plot of monthly groundwater level variation versus year.

Previous researches reveal that groundwater exploitation is the major factor inducing land subsidence^1,2,6^. Accordingly, the groundwater level records are selected as one of the input features. The well depth of the multi-layer groundwater level monitoring wells at STES, YCES, and NLPS is 134 m, 90 m, 189 m, respectively. The monthly groundwater level variation is calculated as follows.

2 $$ \Delta G = G_{i} - G_{i - 1} , $$

where $\Delta G$ denotes the monthly groundwater level variation, $G_{i}$ denotes the groundwater level at the *i*-th month, and $G_{i - 1}$ denotes the groundwater level at the (*i*–1)-th month. Figure 7a,b,c illustrate the plot of monthly groundwater level variation at STES, YCES, and NLPS, respectively. The groundwater level data show obvious seasonal changes in wet and dry seasons every year.

#### Monthly electricity consumption of managed wells

Land subsidence is a recognized consequence of excessive groundwater exploitation, making the investigation of groundwater usage a critical aspect of this study. However, data directly related to well discharge and groundwater usage are unavailable. Consequently, we conducted a correlation analysis to explore the relationship between pumping rate and the electricity consumption of managed wells. For this analysis, we focused on a total of 107 wells located within a 2500 m radius of the STES. It is found that within a 2500 m radius of the STES, the pumping rate and electricity consumption exhibit a high positive correlation, with a correlation coefficient of 0.97. This analysis demonstrates a strong positive association between pumping rate and electricity consumption. Accordingly, we employ electricity consumption by wells as a proxy indicator for estimating pumping rate, which in turn represents groundwater usage.

**Figure 8:**
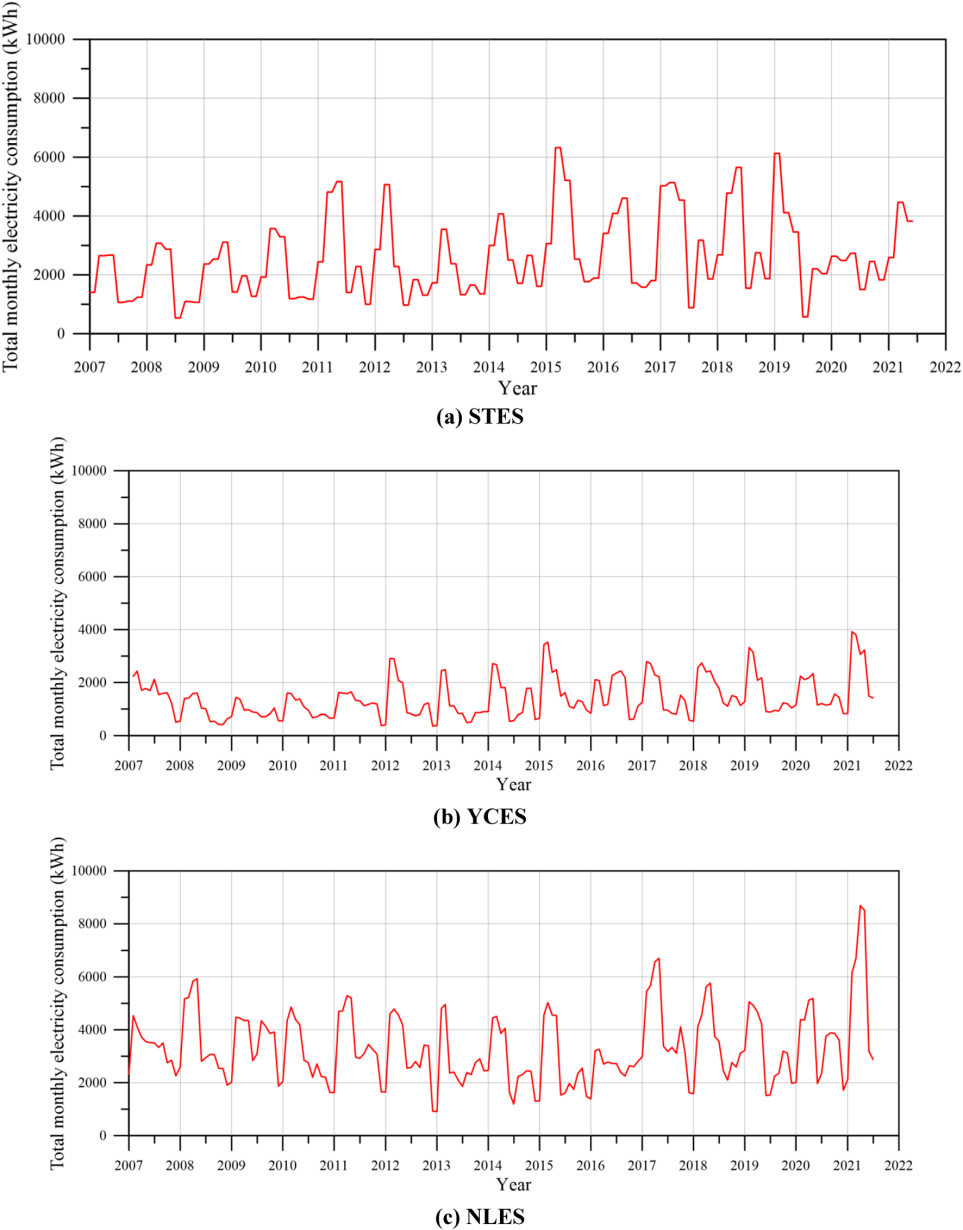
Plot of total monthly electricity consumption versus year.

The monitored data of electricity consumption for 2017 to 2021 for the managed wells within the 250 m radius of each MLCW were analyzed. Data of the electricity consumption are collected from 39, 18, 27 managed wells at STES, YCES, and NLPS within the 250 m buffer region, respectively. Figure 8a,b,c illustrate the plot of total electricity consumption of managed wells for each month versus year at STES, YCES, and NLPS, respectively. Results of total electricity consumption of managed wells show obvious seasonal changes in wet and dry seasons every year. Based on the total electricity consumption of managed wells, the monthly electricity consumption variation of managed wells can be evaluated as

3 $$ \Delta E = E_{i} - E_{i - 1} , $$

where $\Delta E$ is the monthly electricity consumption variation, $E_{i}$ is the electricity consumption at the *i*-th month, and $E_{i - 1}$ presents the electricity consumption at the (*i*–1)th month. The electricity consumptions of wells in the buffer region are composed of time series electricity consumption recorded on a selected managed wells distributed over the study area.

#### Monthly precipitation

Precipitation plays a pivotal role in influencing land subsidence. Positive values for precipitation variation indicate an increase in rainfall, which can contribute to higher groundwater recharge. This may lead to reduced land subsidence. Negative values for precipitation variation signify a decrease in rainfall, potentially resulting in less groundwater recharge and potentially more significant land subsidence. Accordingly, the monthly precipitation records is selected as one of the input features.

**Figure 9:**
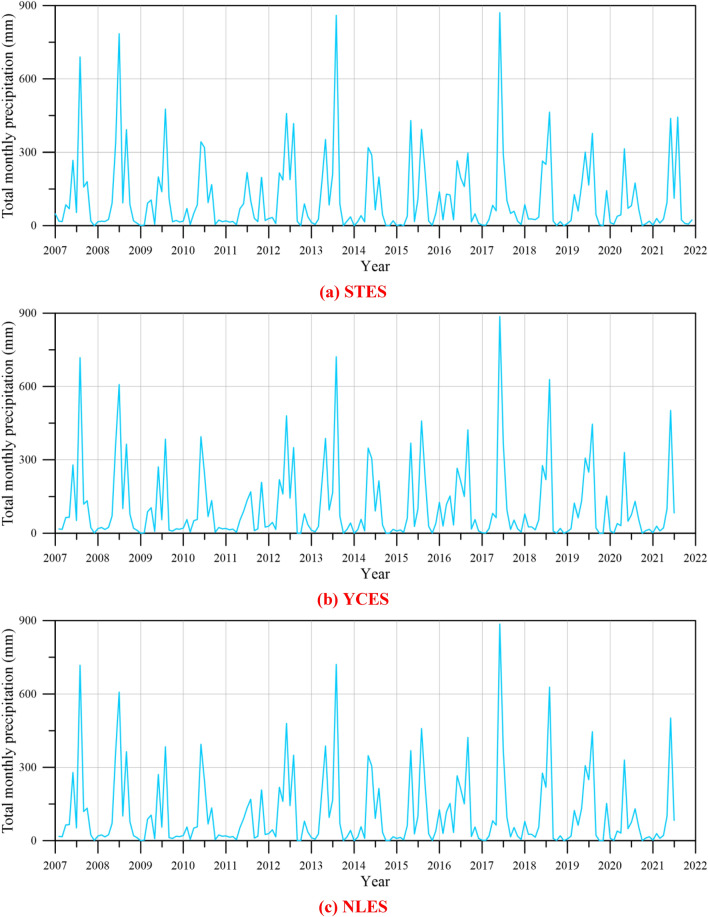
Plot of total monthly precipitation variation versus year.

The total monthly precipitation data are from the Central Weather Bureau. Figure 9a,b,c, illustrate the plot of total precipitation for each month versus year at STES, YCES, and NLPS, respectively. From June to September, there is a concentration of rainfall, which represents around 80% of the total annual precipitation. The variation of average monthly precipitation was calculated as follows:

4 $$ \Delta R = R_{i} - R_{i - 1} , $$

where $\Delta R$ denotes the variation of average monthly precipitation, $R_{i}$ denotes the average monthly precipitation data at the *i*-th month, and $R_{i - 1}$ denotes the average monthly precipitation data at the (*i*–1)^th^ month.

#### Percentage of agricultural land use

Increased agricultural activity may have an impact on land subsidence, especially in areas with extensive irrigation practices. Figure 4 provides a thematic map illustrating the land use inventory. The study area is segmented into several distinct land use categories including agricultural land, aquacultural land use, livestock land use, manufacturing land use, and other regulated districts^31^, as depicted in Fig. 4. This depiction highlights that agriculture predominantly characterizes the land use in the study area, which includes STES, YCES, and NLPS.

In this study, we computed the percentage of agricultural land use within a 250 m radius of each MLCW. To perform this calculation, we utilized the buffer analysis tool in ArcGIS, which generates buffer polygons around input features at a specified distance for spatial analysis. This analysis allowed us to determine the proportion of agricultural land within the designated area. The percentage of agricultural land use is defined as follows.

5 $$ P_{A} = \frac{{A_{a} }}{{A_{T} }}, $$

where $P_{A}$ is percentage of agricultural land use, $A_{a}$ is area of agricultural land use in the division unit, and $A_{T}$ is total area of the unit.

#### Percentage of fine-grained soil

Fine-grained soils may be susceptible to compaction when subjected to excessive groundwater extraction^6,11,13^. This compaction may result in land subsidence. The percentage of fine-grained soil were generated from the borehole data of the CGS and WRA of Taiwan. In accordance with the Unified Soil Classification System, fine-grained soils are characterized by the fact that 50% or more of their particles pass through the No. 200 sieve^32^. Fine-grained soils encompass three distinct types: fine sand, silt, and clay. The percentage of fine-grained soil is determined by calculating the ratio of the combined thickness of fine sand, silt, and clay layers to the total drilling depth^32^. The percentage of fine-grained soil is evaluated using the following equation

6 $$ P_{F} = \frac{{H_{F} }}{{H_{T} }}, $$

where $P_{F}$ is the percentage of fine-grained soil, $H_{F}$ is the soil thickness of fine-grained soil, and $H_{T}$ is the total soil thickness.

#### Length of the average maximum drainage path

To describe the deformation of fine-grained soils under consolidation, it is crucial to consider the length of the average maximum drainage path^6,11,13^. Considering the top and bottom drainage conditions for the soil layer, the length of average maximum drainage path is defined as the average drainage path length^32^, which can be expressed as follows.

7 $$ H_{dr} = \frac{1}{n}\sum\limits_{i = 1}^{n} {(H_{if} /2)} , $$

where *n* denotes the number of fine-grained soil layer, $H_{dr}$ denotes the length of the average maximum drainage path during compaction and $H_{if}$ denotes the soil thickness of fine-grained soil.

## Methodology

### Principal component analysis (PCA)

In the Choshui Delta, extensive and long-term environmental monitoring has been conducted over the years, encompassing groundwater level observations, rainfall measurements, and land subsidence monitoring, resulting in a substantial amount of available data^8,10–12,14^. The primary objectives include gaining insights into groundwater hydrology, meteorological hydrology, as well as the compressional characteristics of subsurface geological formations and the land subsidence patterns of various soil layers at different depths. Due to data that can potentially serve as input factors, it becomes essential to identify the relevant and meaningful factors for neural networks. To address this challenge, the utilization of PCA emerges as a statistical technique that effectively reduces data dimensionality while retaining the crucial information.

Our approach is designed to capture the intricate interactions among these variables within the context of land subsidence.

We propose a novel algorithm that leverages a multi-factorial perspective to accurately reconstruct the missing time-varying land subsidence data. By considering eight influential factors, our method seeks to capture the intricate interplay among these variables in the land subsidence process. Utilizing PCA, we ascertain the significance of these influencing factors and their principal components in relation to land subsidence. To reconstruct the absent time-dependent land subsidence data using PCA-derived principal components, we employ the backpropagation neural network.

Furthermore, the PCA results can influence the selection of input variables for the backpropagation neural network. By identifying the principal components that explain the most variance in the data, we can choose principal components as inputs for the neural network. This selection can enhance the network's training and predictive performance.

The PCA was carried out to obtain a set of principal components (PCs) that are linearly uncorrelated, defined as

8 $$ {\mathbf{AX}} = {\mathbf{X}}\lambda , $$

where *λ* is the eigenvalue, **X** represents the input data, and **A** represents a matrix. Using the linear transformation, we obtain the following equations:

9 $$ {\mathbf{AE}} = {\mathbf{E}}\lambda , $$

10 $$ {\mathbf{Y}} = {\mathbf{E}}^{\prime}{\mathbf{X}}, $$

where **E** is the PC (eigenvector), and **Y** is the transformed variable. Equations (6) and (7) can be rewritten as

11 $$ {\mathbf{A}}_{m \times m} {\mathbf{E}}_{m \times q} = {\mathbf{E}}_{m \times q} \lambda_{q \times q} , $$

12 $$ {\mathbf{Y}}_{q \times n} = {\mathbf{E}}^{\prime}_{q \times m} {\mathbf{X}}_{m \times n} , $$

where *n* is the features number. According to the above transformation, the dimensionality reduction is achieved and the dimensionality of original input data **X** was reduced from *m* to *q*. The original **X** was converted into the transformed variable **Y** by using the PC as the weights. Therefore, the following equations are achieved

13 $$ {\mathbf{S}}_{m \times m} {\mathbf{E}}_{m \times q} = {\mathbf{E}}_{m \times q} {{\varvec{\uplambda}}}_{q \times q} , $$

14 $$ {\mathbf{Y}}_{q \times n} = {\mathbf{E}}^{\prime}_{q \times m} {\mathbf{X}}_{m \times n} , $$

where **S** is the covariance matrix defined as

15 $$ {\mathbf{S}}_{m \times m} = \frac{1}{n - 1}{\mathbf{X}}_{m \times n} {\mathbf{X}}^{\prime}_{n \times m} , $$

After computing the covariance matrix, the correlations are then identified. Equations (10) and (11) are rewritten as following equations once the reduction of dimensionality is unnecessary,

16 $$ {\mathbf{S}}_{m \times m} {\mathbf{E}}_{m \times m} = {\mathbf{E}}_{m \times m} \lambda_{m \times m} , $$

17 $$ {\mathbf{Y}}_{m \times n} = {\mathbf{E}}^{\prime}_{m \times m} {\mathbf{X}}_{m \times n} , $$

Finally, the eigenvectors and eigenvalues of the covariance matrix are computed to identified the PC^13,33^.

In this study, PCA serves as a preprocessing step in this study to assess the relationships between influencing factors and land subsidence, thereby enhancing data analysis and modeling. Its primary roles include the identification of influential factors, dataset simplification, and the potential enhancement of subsequent BPNN performance. Moreover, PCA is a linear dimensionality reduction technique that is primarily designed to capture linear relationships between variables. PCA works by finding linear combinations of the original variables that maximize the variance in the data. It is noted that PCA has limitations to capture non-linear relationships between subsidence and predictor variables. It is important to clarify that PCA itself does not directly resolve the issue of filling data gaps. Instead, it assists in understanding the underlying data structure and selecting the most relevant variables for modeling, which can indirectly improve the handling of missing data. PCA provides a comprehensive view of the data's internal structure, making it suitable for scenarios where variables may have intricate interactions. While correlation analysis is valuable, it may not capture all aspects of data complexity.

### Artificial neural network

**Figure 10:**
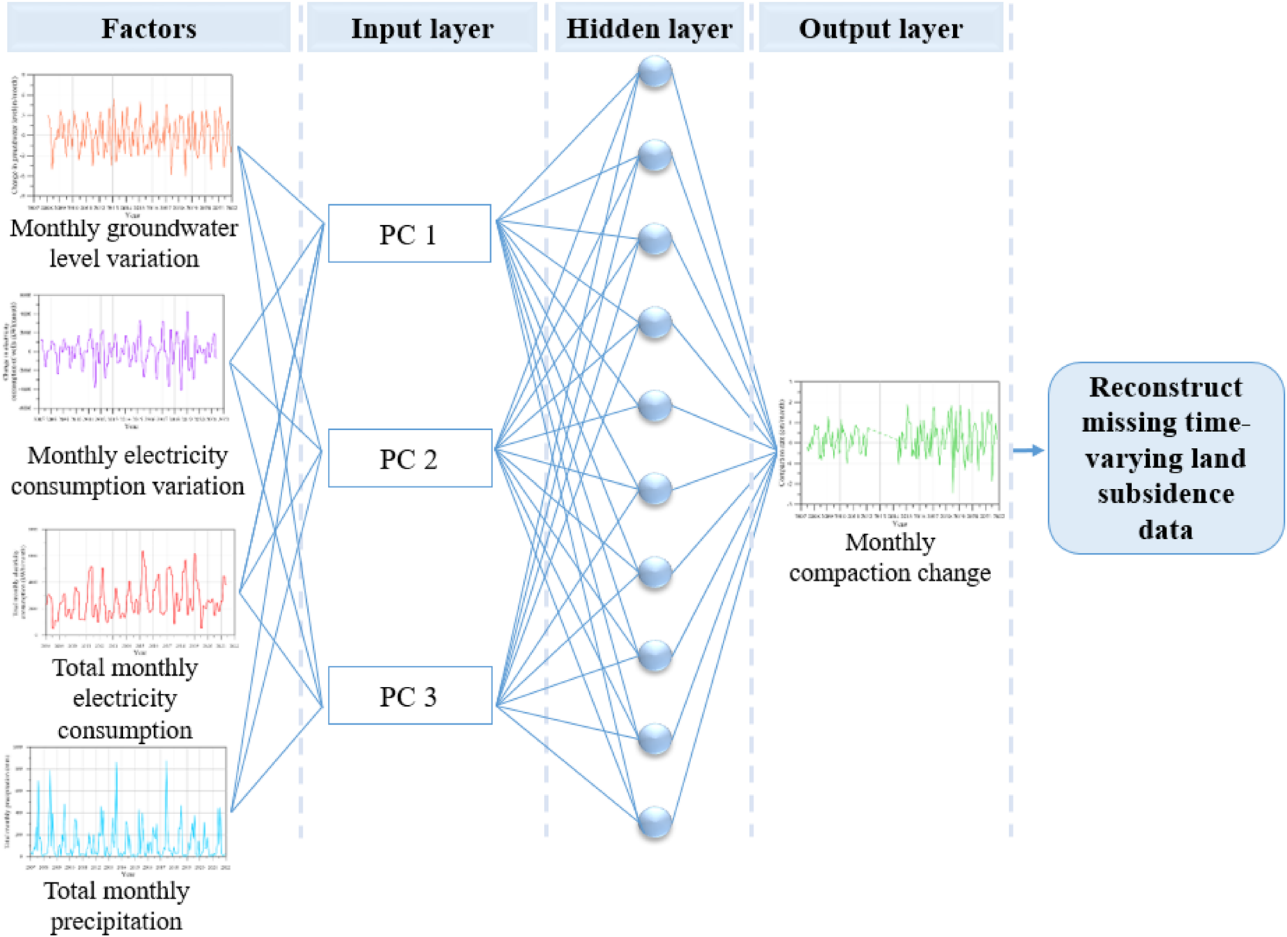
Structure of the proposed BPNN.

The spatiotemporal modeling of subsidence integrates the spatial characteristics and temporal nonlinearity of land subsidence. The overall framework comprises two main aspects: the construction of a spatiotemporal dataset and the modeling of land subsidence in the spatiotemporal domain^15,16^. The spatiotemporal dataset is constructed by the time series input features obtained by WRA leveling surveys and MLCWs. The spatiotemporal modeling involves three components: temporal evolution modeling, spatial correlation analysis, and spatiotemporal integration. Finally, the model is trained by adopting a substantial amount of time series data (February 2008 to February 2012 and April 2014 to June 2021) on land subsidence collected in Yunlin County. The structure of a basic BPNN is shown in Fig. 10. For time series prediction of land subsidence from groundwater withdrawals using artificial neural network (ANN)^20,28^, the training phase and the achieved outcomes are characterized as

18 $$ y_{i} = \phi (X_{j} ) = \left[ {\beta_{oj} + \sum\limits_{i = 1}^{I} {\left( {\beta_{ij} x_{i} } \right)} } \right], $$

19 $$ Z_{k} = \phi (Y_{k} ) = \left[ {\beta_{ok} + \sum\limits_{j = 1}^{J} {\left( {\beta_{kj} y_{i} } \right)} } \right], $$

where $y_{i}$ is the hidden layer, $Z_{k}$ is the output layer, $\phi$ is the activation function, $X_{j}$ and $Y_{k}$ are the temporarily numerical results before utilizing the activation function, $x_{i}$ is the input layer, $\beta_{oj}$ and $\beta_{ok}$ are the bias weight, $\beta_{ij}$ and $\beta_{kj}$ are the weights of the connections. The activation function in this study was hyperbolic tangent sigmoid function. The hidden and output layers can be designated as

20 $$ y_{i} = \phi (X_{j} ) = \phi \left( {\frac{1}{{1 + e^{{ - X_{j} }} }}} \right), $$

21 $$ Z_{k} = \phi (Y_{k} ) = \phi \left( {\frac{1}{{1 + e^{{ - Y_{k} }} }}} \right), $$

The following error function (*EF*) is applied for error backpropagation weight training

22 $$ EF = \frac{1}{2}\sum\limits_{k = 1}^{K} {\left( {\varpi_{k}^{2} } \right)} = \frac{1}{2}\sum\limits_{k = 1}^{K} {\left( {t_{k} - z_{k} } \right)^{2} } , $$

where $\varpi_{k}$ and $t_{k}$ are the error and target value for each node of the output. The objective is to minimize the above error function. The adjustment of weight between the hidden and output layers is

$$ \Delta \beta_{kj} = \mu \times y_{i} \times \delta_{k} , $$

where $\mu$ presents the learning rate ranging from 0 to 1. The updated weight herein is then calculated by using the following equation:

$$ \beta_{kj} {(}\upsilon + {1)} = \beta_{kj} (\upsilon ) + \Delta \beta_{kj} (\upsilon ), $$

where $\upsilon$ presents the iteration number. The gradient of *EF* between the input and hidden layers is

25 $$ \frac{\partial EF}{{\partial \beta_{ij} }} = \sum\limits_{k = 1}^{K} {\frac{\partial EF}{{\partial z_{k} }}} \frac{\partial z}{{\partial Y_{k} }}\frac{{\partial_{k} }}{{\partial y_{i} }} \times \frac{{\partial y_{i} }}{{\partial X_{j} }} \times \frac{{\partial X_{j} }}{{\partial \beta_{ij} }} = - \Delta_{j} x_{i} , $$

26 $$ \Delta_{j} = \phi ^{\prime}(X_{j} )\sum\limits_{k = 1}^{K} {\left( {\delta_{k} \beta_{kj} } \right)} . $$

The updated weighting can be expressed as

27 $$ \Delta \beta_{ij} = \eta \times x_{i} \times \Delta_{j} , $$

28 $$ \beta_{ij} (\upsilon + 1) = \beta_{ij} (1) + \Delta \beta_{ij} (\upsilon ). $$

Two evaluation metrics were utilized to assess the performance of the proposed method. Firstly, the root mean square error (RMSE), a widely recognized metric in predictive modeling. RMSE quantifies the average discrepancy between the predicted values and the actual observed data.

In this study, eight influential factors, encompassing monthly groundwater level variation, monthly electricity consumption variation, variation of average monthly precipitation, percentage of agricultural land use, percentage of fine-grained soil, length of the average maximum drainage path, total monthly electricity consumption, and total monthly precipitation, were included in the PCA. As a result, we employ PCA to assess the relationship between these eight influential factors and land subsidence. Utilizing PCA, we ascertain the significance of these influencing factors and their principal components in relation to land subsidence. To reconstruct the absent time-dependent land subsidence data using PCA-derived principal components, we employ the backpropagation neural network.

## Results

The PCA is initially utilized to assess the relationship between the influencing factors and land subsidence. To reconstruct the missing time-varying land subsidence data based on the factors identified through PCA, we employ the BPNN. Detailed findings from this analysis are elaborated in the following sections.

### Investigating the dominant factors and generating principal components

In this study, we adopt the PCA to examine the dominant factors effecting subsidence and generating principal components. The PCA results can be used to the input variables for the BPNN. By identifying the principal components that explain the most variance in the data, we can choose the dominant factors affecting land subsidence as inputs for the neural network. This selection can enhance the network's training and predictive performance. The dataset of three MLCWs at the STES, YCES, and NLPS from 2008 to 2021 were adopted.

As listed in Table 2, eight influential factors, denoted as factors 2 through 9, encompassing monthly groundwater level variation, monthly electricity consumption variation, variation of average monthly precipitation, percentage of agricultural land use, percentage of fine-grained soil, length of the average maximum drainage path, total monthly electricity consumption, and total monthly precipitation, were included in the PCA. Consequently, we applied PCA to evaluate the relationship between these eight influential factors and factor 1, representing monthly compaction change, which is indicative of land subsidence.

**Table 2:** The covariance matrix for the three MLCWs at the STES, YCES, and NLPS.

	Factor 1	Factor 2	Factor 3	Factor 4	Factor 5	Factor 6	Factor 7	Factor 8	Factor 9
Factor 1	1.00	0.75	0.61	0.15	0.08	− 0.05	− 0.08	0.48	0.29
Factor 2	0.75	1.00	0.69	− 0.10	0.01	− 0.01	− 0.01	0.34	0.50
Factor 3	0.61	0.69	1.00	− 0.02	0.01	− 0.01	− 0.01	0.50	0.47
Factor 4	0.15	− 0.10	− 0.02	1.00	0.00	0.00	0.00	0.12	− 0.62
Factor 5	0.08	0.01	0.01	0.00	1.00	− 0.80	− 0.84	0.48	0.01
Factor 6	− 0.05	− 0.01	− 0.01	0.00	− 0.80	1.00	0.35	− 0.23	− 0.01
Factor 7	− 0.08	− 0.01	− 0.01	0.00	− 0.84	0.35	1.00	− 0.55	− 0.01
Factor 8	0.48	0.34	0.50	0.12	0.48	− 0.23	− 0.55	1.00	0.10
Factor 9	0.29	0.50	0.47	− 0.62	0.01	− 0.01	− 0.01	0.10	1.00
Factor 1	Monthly compaction change								
Factor 2	Monthly groundwater level variation								
Factor 3	Monthly electricity consumption variation								
Factor 4	Variation of average monthly precipitation								
Factor 5	Percentage of agricultural land use								
Factor 6	Percentage of fine − grained soil								
Factor 7	Length of average maximum drainage path								
Factor 8	Total monthly electricity consumption								
Factor 9	Total monthly precipitation								

We first evaluate the relationship of the factors with land subsidence using the PCA. By calculating the correlation coefficient matrix, as listed in Table 2, factor 1 is the monthly compaction change, which is positively correlated with the factor 2 (monthly groundwater level variation) and factor 3 (monthly electricity consumption variation of managed wells). The correlation of the factor 2 is the highest, which is 0.75, indicating the variation of land subsidence is highly related to the fluctuation of groundwater level. Additionally, factor 3 (monthly electricity consumption variation) had a correlation coefficient of 0.61 with factor 1 (monthly compaction change), showing that land subsidence is significantly related to electricity consumption fluctuation.

Furthermore, results indicate that factor 8 (total monthly electricity consumption) and factor 9 (total monthly precipitation) had a moderate positive correlation, with correlation coefficients of 0.48 and 0.29, respectively, with factor 1 (monthly compaction change). Based on the PCA results, the primary factors influencing subsidence are identified as factor 2 (monthly groundwater level variation), factor 3 (monthly electricity consumption variation), factor 8 (total monthly electricity consumption) and factor 9 (total monthly precipitation).

Therefore, the above four factors have been selected for determining principal components in the PCA for the STES, YCES, and NLPS.

Table 3 lists the component loading values in the PCA for the STES, YCES, and NLPS, allowing us to assess the correlation between each factor and the PCs. From Table 3, it is found that factor 2 (monthly groundwater level variation) and factor 3 (monthly electricity consumption variation) for the STES, YCES, and NLPS exhibit correlations of 0.55 or higher with PC 1. Similarly, factor 8 (total monthly electricity consumption) and factor 9 (total monthly precipitation) for the STES, YCES, and NLPS also have correlations of 0.4 or higher with PC1. Additionally, it appears that for the STES, YCES, and NLPS, PC 2 is primarily influenced by factor 8 (total monthly electricity consumption).

**Table 3:** The values of component loading in the PCA for the STES, YCES, and NLPS.

		PC 1	PC 2	PC 3	PC 4
STES					
Factor 2	Monthly groundwater level variation	0.54	− 0.23	− 0.70	− 0.41
Factor 3	Monthly electricity consumption variation	0.59	0.16	− 0.07	0.79
Factor 8	Total monthly electricity consumption	0.42	0.72	0.33	− 0.43
Factor 9	Total monthly precipitation	0.44	− 0.63	0.63	− 0.14
YCES					
Factor 2	Monthly groundwater level variation	0.54	− 0.24	0.67	− 0.44
Factor 3	Monthly electricity consumption variation	0.59	0.16	0.10	0.79
Factor 8	Total monthly electricity consumption	0.40	0.75	− 0.32	− 0.41
Factor 9	Total monthly precipitation	0.45	− 0.59	− 0.66	− 0.13
NLPS					
Factor 2	Monthly groundwater level variation	0.55	0.14	− 0.66	− 0.49
Factor 3	Monthly electricity consumption variation	0.58	− 0.01	− 0.12	0.81
Factor 8	Total monthly electricity consumption	0.50	0.46	0.69	− 0.25
Factor 9	Total monthly precipitation	0.34	− 0.88	0.26	− 0.21

As listed in Table 4, it provides information on eigenvalues and their contributions to the PCs. The representativeness of each PC in explaining the entire dataset is determined by its contribution rate. Upon analyzing the eigenvalues and contribution rates of the factors, it becomes evident that the cumulative contribution of the first three PCs for the STES, YCES, and NLPS all exceed 93%. This observation implies that the first three PCs collectively account for over 90% of the data, indicating a significant level of representativeness. These three PCs are subsequently employed as input variables for the subsequent BPNN analysis.

**Table 4:** Eigenvalue, rate of contribution and cumulative contribution of the principal components.

	Eigenvalue	Rate of contribution (%)	Cumulative contribution (%)
STES			
PC 1	2.39	59.79	59.79
PC 2	0.92	22.92	82.71
PC 3	0.45	11.28	93.99
PC 4	0.24	6.01	100.00
YCES			
PC 1	2.4	59.6	59.6
PC 2	0.9	23.4	83.0
PC 3	0.4	11.2	94.2
PC 4	0.2	5.8	100.0
NLPS			
PC 1	2.5	62.6	62.6
PC 2	0.9	21.8	84.4
PC 3	0.4	9.3	93.7
PC 4	0.3	6.3	100.0

### Reconstructing the missing subsidence data using the BPNN

According to the WRA, the subsidence data from the MLCWs installed at STES, YCES, and NLPS are not available from March 2012 to March 2014. The missing time varying subsidence data are reconstructed in this study using the BPNN. The proposed methodology was applied to reconstruct the missing subsidence data at STES, YCES, and NLPS in Yunlin County. In the BPNN network, the discontinuity in the measured subsidence data is first recovered from the available data. The series with minor gaps of 24 months (from March 2012 to March 2014) is filled. These completed series are then carried out to predict other time series subsidence data. Predictive data are based on learning complete data from the three MLCWs installed at STES, YCES, and NLPS. Parameters for the BPNN model are listed in Table 5.

**Table 5:** Parameters used for the BPNN model.

	Initial value	Stopping criteria	Target value
Epoch	0	9	1000
Elapsed time	–	1.66 s	–
Performance	1.09 $\times$ 10^−3^	6.6 $0\times$ 10^−3^	0
Gradient	6.00 $\times$ 10^−1^	1.7 $0\times$ 10^−2^	10^−7^
mu	10^−3^	10^−4^	10^10^
Validation checks	0	6	6

We first train the BPNN network using the monitored subsidence data spanning a 14-year period from 2008 to 2021 (specifically, February 2008 to February 2012 and April 2014 to June 2021). After the training phase of the BPNN, we test these subsidence data to recover the observations before extending the prediction to complete the sequence. In the BPNN network, the monitored subsidence data spanning from February 2008 to February 2012 and from April 2014 to June 2021 were randomly divided into training, testing, and validation datasets, with an allocation ratio of 70%, 15%, and 15%, respectively. All subsequent analyses related to hidden layers utilize a consistent count of 10. The Levenberg–Marquardt algorithm is used in the training phase of the BPNN. The PCs, as listed in Table 3, have been selected as input variables for the BPNN. RMSE value was calculated using the testing dataset to evaluate the impact of rainfall on the BPNN model's performance.

The predictive accuracy of the BPNN is summarized in Table 6. Three scenarios of input variables, including first: PC1, second: PC1 and PC2, and third: PC1, PC2 and PC3 are considered. Considering all three PCs as input variables for computing the RMSE of the testing dataset at STES, YCES and NLPS, it appears that the scenario with the consideration of all three PCs as input variables achieves great accuracy for three sites.

**Table 6:** RMSE for the testing dataset using the PCs in the BPNN.

Site	RMSE (PC 1)	RMSE (PC 1 and PC2)	RMSE (PC 1, PC 2 and PC3)
STES	0.35	0.30	0.30
YCES	0.36	0.20	0.13
NLPS	0.07	0.40	0.13

**Figure 11:**
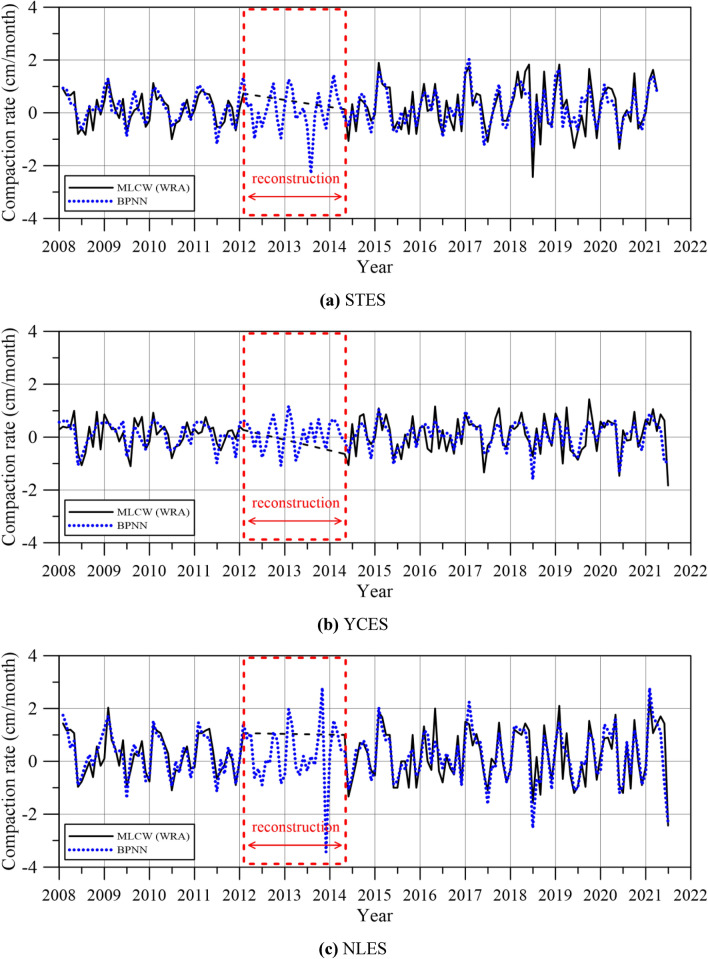
Reconstruction of missing compaction data using the three PCs in the BPNN.

**Figure 12:**
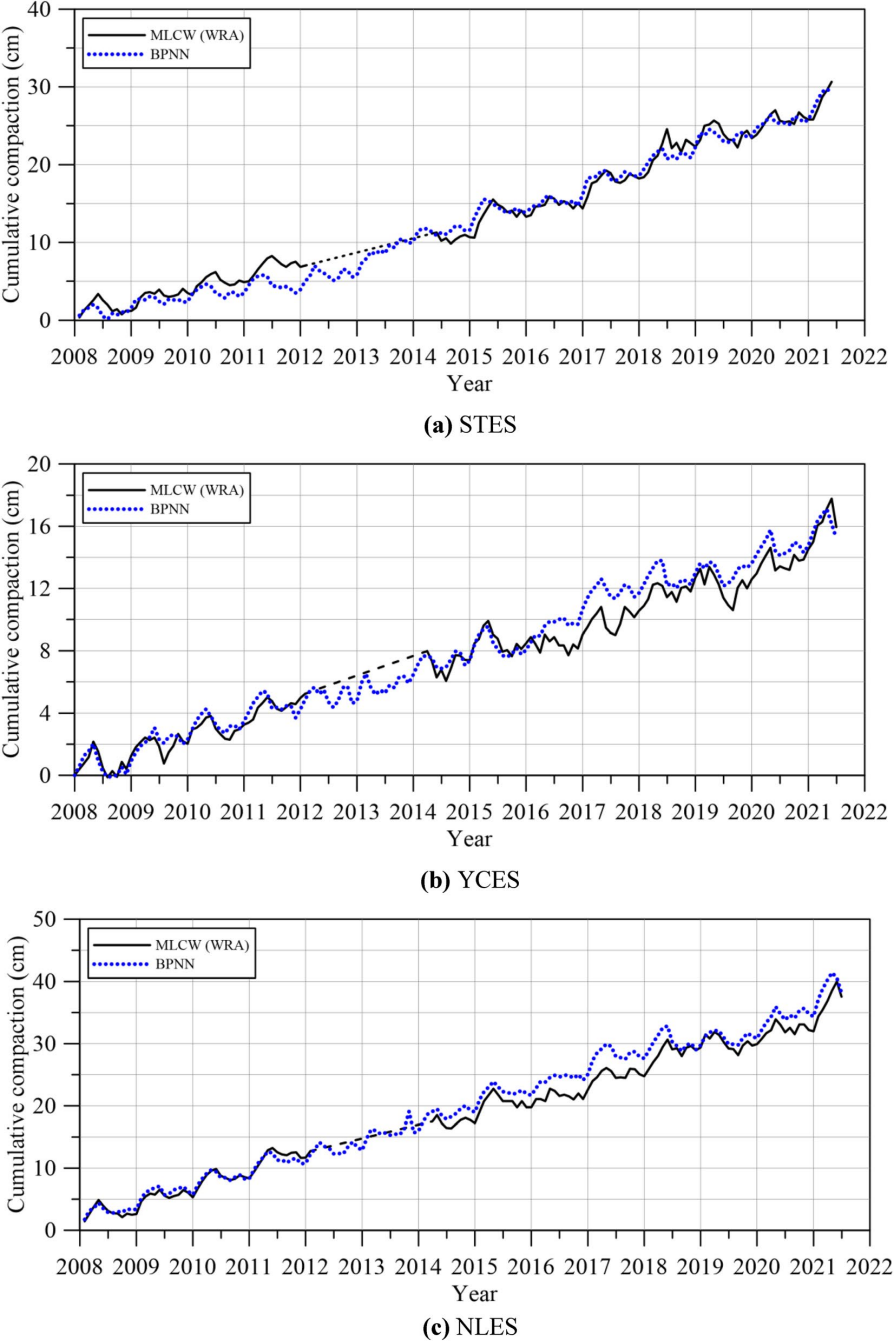
Comparison of results with observed data from the WRA ^34^.

Figure 11 illustrates the reconstruction of missing compaction data using the BPNN. It reveals that employing three PCs as input variables for the BPNN can successfully reconstruct missing compaction data. Consequently, this study proceeded and generated a graphical representation of cumulative subsidence over the years. As depicted in Fig. 12, we compare the predicted subsidence data obtained using the BPNN model with the monitored subsidence data provided by the WRA^34^. Results reveal that good agreement can be obtained between the predictive results generated by the proposed BPNN model and the monitored subsidence data from the WRA^34^.

## Discussion

The assessment of the relationship between influencing factors and land subsidence in this study begins with PCA. In the PCA, eight influential factors, encompassing monthly groundwater level variation, monthly electricity consumption variation, variation of average monthly precipitation, percentage of agricultural land use, percentage of fine-grained soil, length of the average maximum drainage path, total monthly electricity consumption, and total monthly precipitation, were included in the PCA. Based on the PCA results, primary factors influencing subsidence are identified as monthly groundwater level variation, monthly electricity consumption variation, total monthly electricity consumption and total monthly precipitation. Therefore, factors encompass variations in groundwater levels, fluctuations in electricity consumption of managed wells, total monthly electricity consumption and total monthly precipitation are selected for determining principal components.

The study's outcomes suggest that the BPNN approach presents itself as a practical and efficient alternative for predicting land subsidence. Its reliance on historical time-series data and the flexibility of not requiring highly detailed hydrogeological parameters make it accessible and applicable in a variety of real-world situations. Furthermore, the model's success in reconstructing missing data enhances its overall utility and robustness.

In summary, the results of the BPNN model demonstrate the effectiveness of the approach in accurately reconstructing subsidence data over extended time periods for these specific sites. This methodology has displayed promise in preserving key features of subsidence data, rendering it highly suitable for the selected areas.

## Conclusions

In this article, we aim to address the challenge of reconstructing missing time-varying land subsidence data in the Choshui Delta, Taiwan. To accomplish this, we propose a novel algorithm that employs a multi-factorial perspective to effectively reconstruct the missing data. We consider eight factors including the groundwater level data, electricity consumption data, precipitation data, land use pattern, sediment type, and drainage path length, which are known to significantly influence land subsidence. Through our analysis, we summarize the key findings as follows:

- To assess the relationship between eight influencing factors and land subsidence, an initial step involves employing PCA. The PCA results reveal that the monthly compaction change exhibits positive correlations with the monthly variation in groundwater level, and the variation in electricity consumption of managed wells. Notably, the correlation with groundwater level variation is found to be the strongest. This indicates that the variability of land subsidence is closely associated with fluctuations in groundwater levels.
- In the BPNN network, the observed results demonstrate good accuracy between the predictions generated by the proposed BPNN model and the historical subsidence data. The results reveal that the reconstruction of missing data using the BPNN approach effectively preserves the key features of the subsidence data.
- Furthermore, the results demonstrate that the proposed neural network model does not require sophisticated soil compaction parameters and complex hydrogeological modeling techniques. This finding highlights the advantages of the BPNN model, especially when time-dependent observations and monitoring data are available.

## Data Availability

The datasets used during the current study available from the corresponding author on reasonable request.
